# C-X-C motif chemokine ligand 13 suppresses osteoclast differentiation via interference with RANKL–RANK interaction

**DOI:** 10.3389/fimmu.2025.1729222

**Published:** 2026-01-09

**Authors:** Trung-Loc Ho, Kun-Tsan Lee, Yu-Hao He, Le Huynh Hoai Thuong, David Achudhan, Wei-Chien Huang, Chih-Yuan Ko, Chun-Lin Liu, Jeng-Hung Guo, Chih-Hsin Tang

**Affiliations:** 1 Graduate Institute of Biomedical Sciences, China Medical University, Taichung, Taiwan; 2 Department of Post-Baccalaureate Medicine, National Chung-Hsing University, Taichung, Taiwan; 3 Department of Orthopedics, Taichung Veterans General Hospital, Taichung, Taiwan; 4 Center for Molecular Medicine, China Medical University Hospital, Taichung, Taiwan; 5 Department of Biomedical Imaging and Radiological Science, China Medical University, Taichung, Taiwan; 6 Department of Physiology and Biomedical Engineering, Mayo Clinic, Rochester, MN, United States; 7 Department of Orthopedic Surgery, China Medical University Hospital, Taichung, Taiwan; 8 Department of Neurosurgery, China Medical University Hospital, Taichung, Taiwan; 9 Department of Pharmacology, School of Medicine, China Medical University, Taichung, Taiwan; 10 Chinese Medicine Research Center, China Medical University, Taichung, Taiwan; 11 Department of Medical Laboratory Science and Biotechnology, Asia University, Taichung, Taiwan

**Keywords:** CXCL13, MAPK, NF-κB, osteoclastogenesis, RANKL

## Abstract

**Introduction:**

Osteoclastogenesis, the differentiation of osteoclasts from monocyte/macrophage precursors, is essential for physiological bone remodeling but contributes to pathological bone loss in arthritis, osteoporosis, and bone metastasis when dysregulated. CXCL13 is a CXC chemokine well recognized for its role in immune regulation; however, its function in osteoclast biology remains undefined. This study aimed to investigate the effects of CXCL13 on RANKL-induced osteoclastogenesis.

**Methods:**

RAW264.7 cells were stimulated with RANKL to induce osteoclastogenesis. Osteoclast formation was evaluated by TRAP and F-actin ring staining, and quantitative real-time PCR (qPCR). GEO bioinformatic analysis revealed gene expression changes during RANKL-induced osteoclastogenesis. Mature osteoclast apoptosis was analyzed by cleaved caspase-3 immunofluorescence staining. MAPK and NF-κB signaling activation was examined by Western blotting and NF-κB luciferase reporter assays. Molecular docking and co-immunoprecipitation were performed to evaluate the interaction between CXCL13 and RANK.

**Results:**

CXCL13 inhibited RANKL-induced osteoclast formation, suppressed osteoclast marker expression, disrupted F-actin ring assembly, and promoted apoptosis in mature osteoclasts. Mechanistically, CXCL13 attenuated MAPK and NF-κB activation and blocked p65 nuclear translocation in a CXCR5-independent manner by competitively interfering with RANKL–RANK binding and downstream RANK–TRAF6 signaling.

**Discussion:**

These findings identify CXCL13 as a novel suppressor of osteoclastogenesis by interfering with RANKL–RANK signaling, unveiling an unrecognized regulatory role in osteoclast biology and suggesting potential therapeutic relevance for bone loss disorders.

## Introduction

Bone homeostasis relies on the dynamic balance between bone-forming osteoblasts and bone-resorbing osteoclasts (1, 2). The coordinated regulation of these two cell types is essential for maintaining skeletal architecture and integrity. Disruption of this balance can lead to pathological bone loss, as seen in osteolytic conditions for instance osteoporosis, arthritis and osteolytic bone metastasis. In these disorders, osteoclast activity becomes dysregulated, resulting in excessive degradation of the bone matrix. This heightened resorptive activity is a major driver of bone loss, contributing to structural weakening, impaired skeletal function, and increased fracture risk.

Osteoclastogenesis is the biological process by which osteoclasts, the primary bone-resorbing cells, differentiate and mature from hematopoietic precursors of the monocyte/macrophage lineage (3). The process is primarily driven by RANKL, which binds to its receptor RANK on osteoclast precursors, initiating a cascade of intracellular signaling (4). In particular, RANKL–RANK interaction promotes the NF-κB activation through MAPK pathways and IKK complex, leading to the transcription of genes essential for osteoclast differentiation and function (5, 6). This includes the upregulation of osteoclast-specific markers for instance *cathepsin K* (*Ctsk*), *acid phosphatase 5, tartrate resistant* (*Acp5*), *matrix metallopeptidase 9* (*Mmp9*) and *nuclear factor of activated T cells 1* (*Nfatc1*), all of which are closely associated with osteoclast differentiation and bone resorptive activity (7, 8). Mature osteoclasts form a specialized actin-rich structure known as the podosome belt or actin ring, which is critical for their attachment to the bone surface and subsequent resorptive function (9–11).

Current treatments for bone loss disorders often focus on inhibiting osteoclast differentiation and function, primarily through targeting the RANK/RANKL axis (12). Among osteoclast-targeted therapies, agents such as Denosumab act by neutralizing RANKL, thereby effectively reducing osteoclast-mediated bone resorption (13). Bisphosphonates, including zoledronic acid and alendronate, are another mainstay of therapy; they inhibit osteoclast function by inducing apoptosis (14). Selective estrogen receptor modulators, such as raloxifene, mimic estrogen’s bone-preserving functions and are used primarily in postmenopausal women (15). Despite these options, long-term use of antiresorptive agents carries risks for instance atypical fractures or osteonecrosis of the jaw, highlighting the need for novel therapeutic strategies with improved safety and efficacy profiles (16).

C-X-C motif chemokine ligand 13 (CXCL13), a member of the CXC chemokine family, was originally identified as B-cell-attracting chemokine 1 due to its function in B-cell migration. CXCL13 is a homeostatic chemokine expressed constitutively by stromal cells in secondary lymphoid tissue-rich regions like the splenic follicle, nodes, and Peyer’s patch germinal centers (17). CXCL13 binds to its receptor CXCR5, thereby facilitating immune cell recruitment and modulating the adaptive immune response (18, 19). Dysregulation of the CXCL13/CXCR5 axis has been implicated in various pathological conditions, including autoimmune diseases (20), cancers (21), and infectious disorders (22–24). Moreover, CXCL13 enhances osteogenic differentiation in bone marrow-derived mesenchymal stem cells (25, 26). Previous findings indicate that CXCL13 inhibition effectively suppresses angiogenesis and mitigates the progression of rheumatoid arthritis (20, 27). In oral squamous cell carcinoma, CXCL13 activates c-Myc, thereby enhancing RANKL generation in stromal and preosteoblastic cells within the tumor–bone microenvironment (28). Moreover, in multiple myeloma, CXCL13 acts as a key mediator within the osteolytic niche, driving macrophage polarization toward an M2-like phenotype facilitating tumor progression (29). These findings highlight the multifaceted role of CXCL13 in both inflammatory and malignant bone disorders, underscoring its potential as a promising therapeutic target. However, the role of CXCL13 in non-cancerous bone loss conditions and its mechanistic involvement in osteoclast regulation remain poorly understood. To address this, we assessed the potential role of CXCL13 in RANKL-induced osteoclastogenesis.

## Material and methods

### Material

The RAW264.7 cell line was sourced from the Bioresource Collection and Research Center (Hsinchu, Taiwan). Recombinant mouse RANKL and CXCL13 proteins were obtained from PeproTech (Rocky Hill, NJ, USA). Antibodies specific to p-ERK (sc-7383), p-JNK (sc-6254), p-p38 (sc-7972), p-IKKα/β (sc-23470-R), p-IκBα (sc-8404), ERK (sc-1647), JNK (sc-7345), p38 (sc-271120), IKKα/β (sc-7607), IκBα (sc-1643), RANK (sc-59981), TRAF6 (sc-8409), and p65 NF-κB (sc-8008) were acquired from Santa Cruz Biotechnology (Dallas, TX, USA). Additionally, the antibody against p-p65 (#3033), cleaved caspase-3 (#9664S) were purchased from Cell Signaling Technology (Danvers, MA, USA).

### Cell culture

RAW264.7 cells were cultured in DMEM (Gibco, Cat# 11965092) supplemented with 10% fetal bovine serum (FBS; HyClone, Cat# SH30071.03), 100 U/mL penicillin, and 100 μg/mL streptomycin (Gibco, Cat# 15140122). The cells were maintained at 37 °C in a humidified incubator with 5% CO_2_.

### Osteoclast differentiation assays

RAW264.7 cells were seeded at a density of 5,000 cells per well in 24-well plates and allowed to adhere overnight in α-MEM supplemented with 10% fetal bovine serum (FBS), 1% penicillin–streptomycin, and L-glutamine as included in the standard α-MEM formulation. Cells were maintained at 37 °C in a humidified incubator containing 5% CO_2_. To examine the effect of CXCL13 on osteoclast differentiation, cells were stimulated from Day 0 to Day 5 with RANKL (50 ng/mL) in the presence or absence of CXCL13 at the indicated concentrations. The culture medium containing RANKL and CXCL13 was refreshed every two days. On Day 5, multinucleated osteoclasts were assessed by tartrate-resistant acid phosphatase (TRAP) staining to quantify osteoclast formation. To determine the effect of CXCL13 on mature osteoclasts, RAW264.7 cells were first differentiated with RANKL (50 ng/mL) alone from Day 0 to Day 5, with medium replacement every two days. On Day 6, mature osteoclasts were exposed to CXCL13 for 24 hours. Following treatment (Day 7), cells were subjected to TRAP staining, F-actin ring staining, podosome belt and cleaved caspase-3 immunostaining to evaluate osteoclast morphology and apoptosis, respectively.

### qPCR assays

RAW264.7 cells were seeded at 1 × 10^5^ cells per well in 6-well plates and cultured to induce osteoclast differentiation with RANKL (50 ng/mL) alone or in combination with dose-dependent CXCL13. Treatments were refreshed every two days for a total of 5 days. Total RNA was isolated from cultured cells using TRIzol™ Reagent (Invitrogen, Cat# 15596018) following the manufacturer’s instructions. cDNA was synthesized from 1 μg of purified RNA using the M-MLV Reverse Transcriptase Kit (Thermo Fisher Scientific, Cat# 28025013, Waltham, MA, USA). Quantitative real-time PCR (qPCR) was performed on a StepOnePlus™ Real-Time PCR System (Applied Biosystems) using gene-specific primers. Gene expression levels were normalized to Gapdh, and relative expression changes were calculated using the 2^-^ΔΔCt method. Primer sequences are provided in **Table 1**.

**Table 1:** Primers were used in this study.

Gene name	Forward primer	Reverse primer
*Mouse Acp5*	5′-ATGGGCGCTGACTTCATCAT-3′	5′-GGTCTCCTGGAACCTCTTGT-3′
*Mouse Ctsk*	5′-AGTAGCCACGCTTCCTATCC-3′	5′-CCATGGGTAGCAGCAGAAAC-3′
*Mouse Mmp9*	5′-GGACCCGAAGCGGACATTG-3′	5′-CGTCGTCGAAATGGGCATCT-3′
*Mouse Nfatc1*	5′-GACCCGGAGTTCGACTTCG-3′	5′-TGACACTAGGGGACACATAACTG-3′
*Mouse Gapdh*	5′-TGTGTCCGTCGTGGATCTGA-3′	5′-TTGCTGTTGAAGTCGCAGGAG-3′

### Western blot assay

RAW264.7 cells were seeded at 1 × 10^5^ cells per well in 6-well plates and cultured under the indicated treatments. Cells were lysed in 100 µL RIPA buffer with protease inhibitors (Roche, USA). Protein concentration was measured using the Pierce™ BCA Kit. Equal protein (30 µg/lane) was separated by 10% SDS-PAGE and transferred to PVDF membranes. Membranes were blocked with 5% non-fat milk in TBS for 1 hour, subsequently incubated with primary antibodies overnight at 4 °C. After washing, HRP-conjugated secondary antibodies were applied (30). Bands were visualized using ECL and imaged with the iBright 1500 system (Thermo Fisher Scientific).

### Cell transfection

RAW264.7 cells were transfected with CXCR5 siRNA or non-targeting control siRNA (ON-TARGETplus, Dharmacon, USA) using Lipofectamine for 24 hours. After transfection, cells were stimulated with RANKL, with or without CXCL13, for 5 days. The culture medium was refreshed every 2 days.

### Luciferase reporter assay

RAW264.7 cells (2 × 10^5^ per well) were seeded in 24-well plates and incubated for 24 hours to allow attachment following previously established protocols (31). Cells were transfected with a κB-luciferase reporter plasmid (Stratagene, USA) using Lipofectamine 2000 (Thermo Fisher Scientific) as per the manufacturer’s instructions. After transfection, cells were treated with RANKL (50 ng/mL) with or without CXCL13 for 24 hours. Luciferase activity was measured using the Dual-Luciferase Reporter Assay System (Promega) on a microplate reader.

### Immunofluorescences assay

RAW264.7 cells were fixed with 3.7% formaldehyde for 15 minutes at room temperature, followed by two PBS washes. Cells were permeabilized with 0.1% Triton X-100 for 15 minutes and blocked with 1% BSA in PBS for 1 hour. To visualize F-actin rings in mature osteoclasts, cells were stained with Phalloidin-Alexa Fluor 488 (Invitrogen, USA) following the manufacturer’s protocol. Cleaved-caspase 3 and NF-κB p65 translocation were detected using primary antibodies (1:200 in 1% BSA) overnight at 4 °C, followed by secondary antibodies conjugated to Alexa Fluor 488 or 594 for 1 hour at room temperature. DAPI (0.1 μg/mL; Sigma-Aldrich, USA) was used for nuclear staining for 15 minutes. Fluorescence images were obtained using the ImageXpress Pico Imaging System (Molecular Devices, USA) and analyzed with the integrated software (8).

### Gene expression omnibus database analysis

The GEO databases GSE225974 and GSE191187 were employed to compare osteoclastogenic gene expression within human and mouse osteoclasts. Central osteoclast marker genes investigated were *Acp5*, *Ctsk*, *Mmp9*, and *Nfatc1*, which are already known to be critical in osteoclast differentiation and function.

### Macromolecular docking and computational modeling

The 3D structures of mouse RANK (PDB ID: 3ME4) and mouse CXCL13 (PDB ID: 5L7M) were obtained from the Protein Data Bank (PDB) (https://www.rcsb.org) (RRID: SCR_012820). The structures were imported into BIOVIA Discovery Studio software (DS2022) (https://www.3ds.com/products/biovia/discovery-studio) (RRID: SCR_015651) for macromolecular docking using the zDOCK module (30). Potential docking poses were selected based on the ZDOCK score, ZRANK score, and clustering analysis. Subsequently, the interacting molecules underwent molecular dynamics simulation procedures, including solvation, the standard dynamics cascade, and dynamics production. The MD simulations were performed for a total of 50 nanoseconds (ns), with data recorded at 10 picosecond (ps) intervals. A total of 5,000 conformations from the production phase were used for trajectory analysis, from which root mean square deviation (RMSD) and root mean square fluctuation (RMSF) values were calculated. RMSD was used to evaluate the overall structural deviation of complex during the simulation relative to the initial structure, thereby indicating whether the system reached stability or underwent large conformational changes. RMSF measured the fluctuation of each residue around its average position throughout the simulation, identifying regions of flexibility or stability. All results were exported and visualized using DS2022 or PyMOL software (RRID: SCR_000305).

### Co-immunoprecipitation assays

RAW264.7 cells were lysed, and 120 μL of lysate (with protease inhibitors) was collected. After sonication (5 cycles, 25 seconds total), samples were divided: 20 μL as input and the remainder for IP. To reduce nonspecific binding, IP samples were precleared with 60 μL of 50% protein A/G-agarose beads (Thermo Fisher, USA) at 4 °C for 1 hour. Supernatants were incubated overnight at 4 °C with 2 μg of anti-RANK antibody. Immune complexes were collected by centrifugation (12,000 rpm, 1 minute, 4 °C) (32). The pellet was resuspended in 20 μL of 5× SDS loading buffer and heated at 95 °C for 5 min to denature proteins before Western blot analysis.

### Statistical analysis

Statistical evaluations were conducted with GraphPad Prism 8.0. Data are expressed as mean ± SD. Two independent group comparisons were analyzed with an unpaired two-tailed Student’s t-test. Multiple group comparisons were analyzed with one-way ANOVA and subsequently with Bonferroni’s *post hoc* testing. Statistical differences were noted when *p* < 0.05.

## Results

### CXCL13 inhibits RANKL-induced osteoclast formation and downregulates osteoclast marker gene production

**Figure 1:**
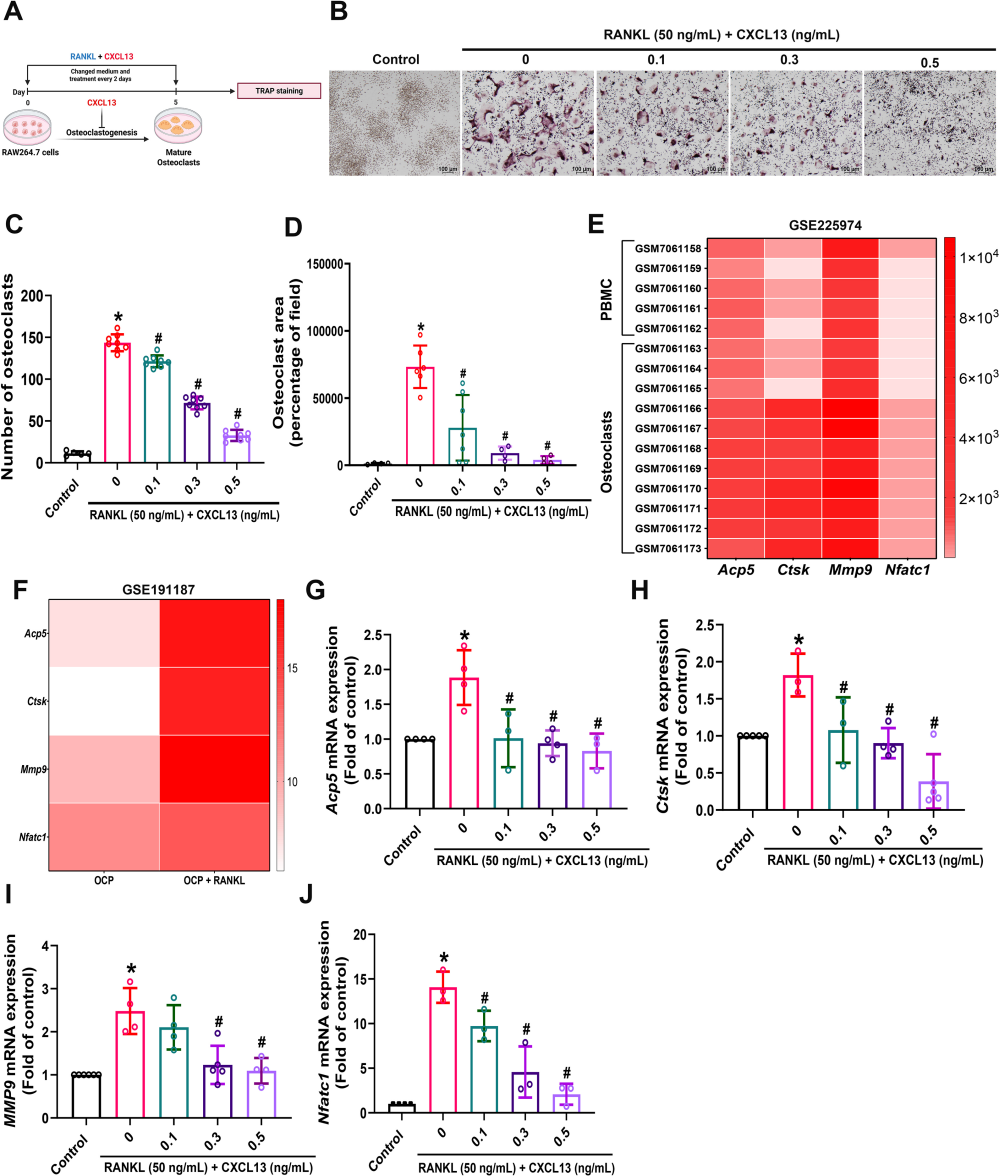
Inhibitory effect of CXCL13 on RANKL-induced osteoclastogenesis. **(A)** RAW264.7 cells were exposed to RANKL (50 ng/mL) and increasing concentrations of CXCL13 (0.1, 0.3, and 0.5 ng/mL) for a period of 5 days. **(B-D)** Osteoclast formation was assessed using TRAP staining, and the number of TRAP-positive multinucleated cells was quantified (scale bar = 100 μm). **(E, F)** GEO dataset analysis revealed the expression profiles of osteoclastogenesis-related markers in both human and mouse osteoclasts. **(G-J)** qPCR analysis was performed to measure changes in gene expression related to osteoclastogenesis following treatment with CXCL13 for 5 days. *p < 0.05 *vs*. control group; #p < 0.05 *vs*. RANKL-treated group. Data represent biological replicates (n ≥ 3). Statistical analysis was performed using one-way ANOVA followed by Tukey’s *post hoc* test.

To assess the role of CXCL13 on osteoclastogenesis, we utilized a standard *in vitro* osteoclast differentiation model based on RANKL-treated RAW264.7 cells cultured for 5 days (**Figure 1A**). The data revealed that CXCL13 inhibited RANKL-induced osteoclast formation in a concentration-dependent manner, as indicated by a significant inhibition in the number of TRAP-positive multinucleated cells (**Figures 1B-D**). To further explore the gene expression profile associated with osteoclastogenesis, we analyzed publicly available datasets from the GEO. In dataset GSE225974, the expression levels of *Acp5*, *Ctsk*, *Mmp9*, and *Nfatc1* were significantly elevated in human osteoclast-like cells compared to peripheral blood mononuclear cells (PBMCs) (**Figure 1E**). Similarly, in dataset GSE191187, these osteoclast-associated genes were markedly elevated in RANKL-treated murine osteoclast precursors (OCPs) relative to untreated controls (**Figure 1F**). Previous studies have also demonstrated that the expression of key osteoclast marker genes, including *Acp5*, *Ctsk*, *Mmp9*, and *Nfatc1*, plays a critical role in regulating osteoclastogenesis (33, 34). Consistent with these transcriptomic data, our qPCR analysis confirmed that RANKL significantly facilitated the expression of *Acp5*, *Ctsk*, *Mmp9*, and *Nfatc1* in RAW264.7 cells, whereas CXCL13 treatment attenuated this induction (**Figures 1G–J**). Collectively, these findings uncover a previously unrecognized role for CXCL13 in suppressing RANKL-induced osteoclastogenesis.

### CXCL13 promotes apoptosis in mature osteoclasts

**Figure 2:**
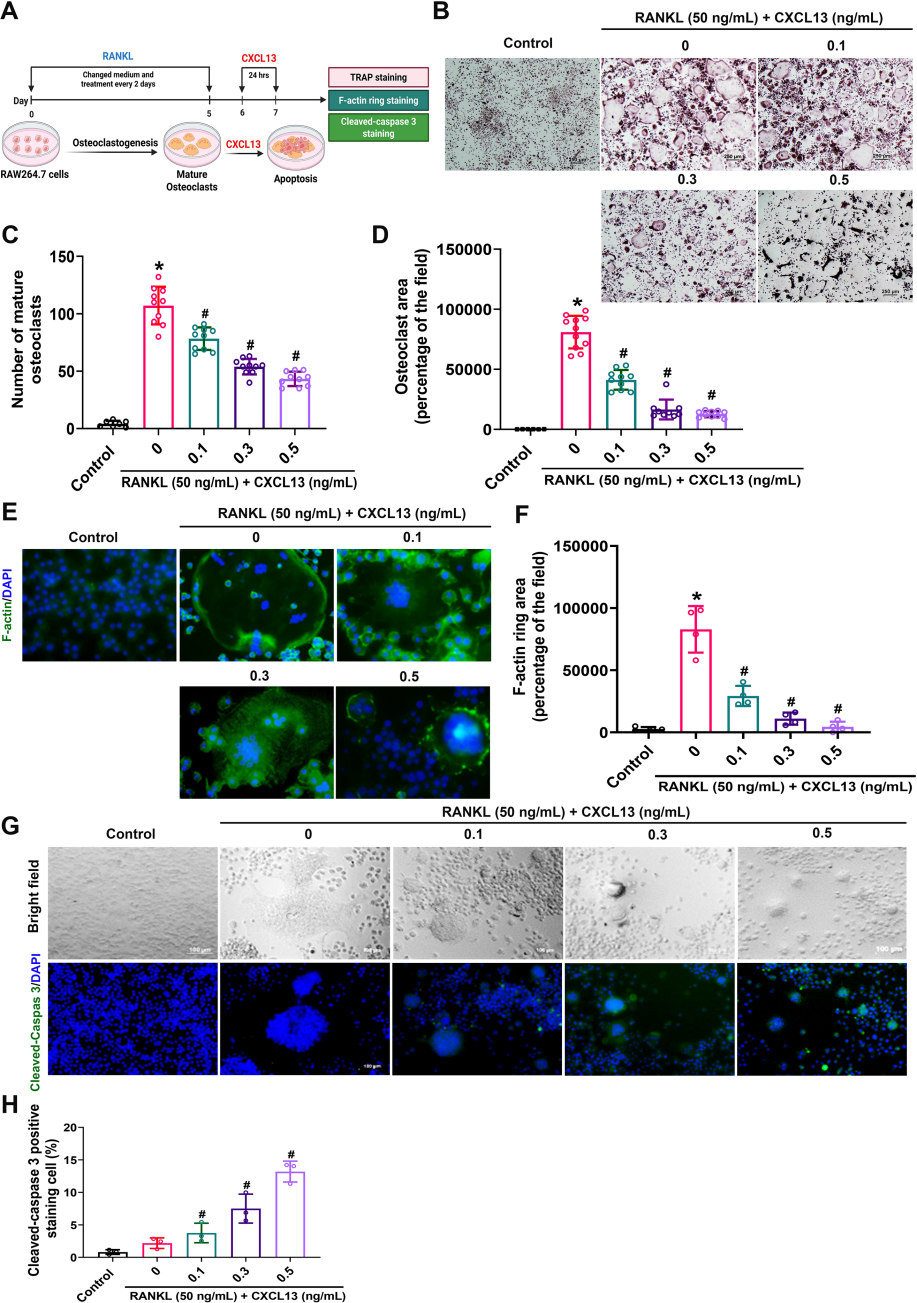
CXCL13 induces cleaved caspase-3-mediated apoptosis in mature osteoclasts. **(A)** RAW264.7 cells were treated with RANKL for 5 days to induce osteoclast differentiation, followed by a 24-hour treatment with CXCL13 **(B-D)** Mature osteoclasts were identified by TRAP staining (scale bar = 250 μm), and their number and area were quantified using ImageJ software. **(E, F)** Immunofluorescence staining was performed to visualize F-actin ring formation, comparing treated and untreated cells, and the ring area was measured. **(G, H)** IF staining also detected cleaved caspase-3 (green) alongside nuclear DAPI staining (blue), with cell morphology visualized under bright-field microscopy (scale bar = 100 μm). The number of cleaved caspase-3 positive cells was quantified using ImageJ. *p < 0.05 *vs*. control. group; #p < 0.05 *vs*. RANKL-treated group. Data represent biological replicates (n ≥ 3). Statistical analysis was performed using one-way ANOVA followed by Tukey’s *post hoc* test.

During osteoclastogenesis, mature osteoclasts form actin ring structures characteristic of their resorptive function and secrete acids and lytic enzymes that facilitate degradation of the mineralized bone matrix (35). To directly examine the role of CXCL13 in mature osteoclasts, RAW264.7 cells were applied with RANKL to promote mature osteoclast formation, followed by CXCL13 treatment for 1 day (**Figure 2A**). TRAP staining indicated that CXCL13 diminished the number and area of mature osteoclasts, as evidenced by a significant inhibition in TRAP-positive multinucleated cells (**Figures 2B–D**). Given that mature osteoclasts rely on specialized cytoskeletal structures, such as podosome belts, for effective bone matrix attachment and resorption, we next examined the impact of CXCL13 on actin cytoskeletal organization. Phalloidin staining was performed to visualize F-actin ring formation, a marker of podosome belt integrity (**Figure 2E**). In the RANKL-only group, well-organized, intact actin rings were observed. In contrast, CXCL13 treatment induced a significant, dose-dependent disruption of the podosome belt structure (**Figure 2F**), suggesting that CXCL13 impairs osteoclast structural organization and potential resorptive capacity. We next examined apoptotic signaling, a key process known to regulate osteoclast lifespan and bone-resorptive function (35). Mature osteoclasts treated with increasing concentrations of CXCL13 showed significant upregulation of cleaved caspase-3, a key marker of apoptosis, compared to the RANKL-only group, as revealed by immunofluorescence analysis (**Figures 2G, H**). Collectively, these data suggest that CXCL13 disrupts cytoskeletal architecture and promotes apoptosis in mature osteoclasts, thereby impairing their survival and resorptive function.

### CXCL13 inhibits RANKL-induced MAPK and NF-κB signaling

**Figure 3:**
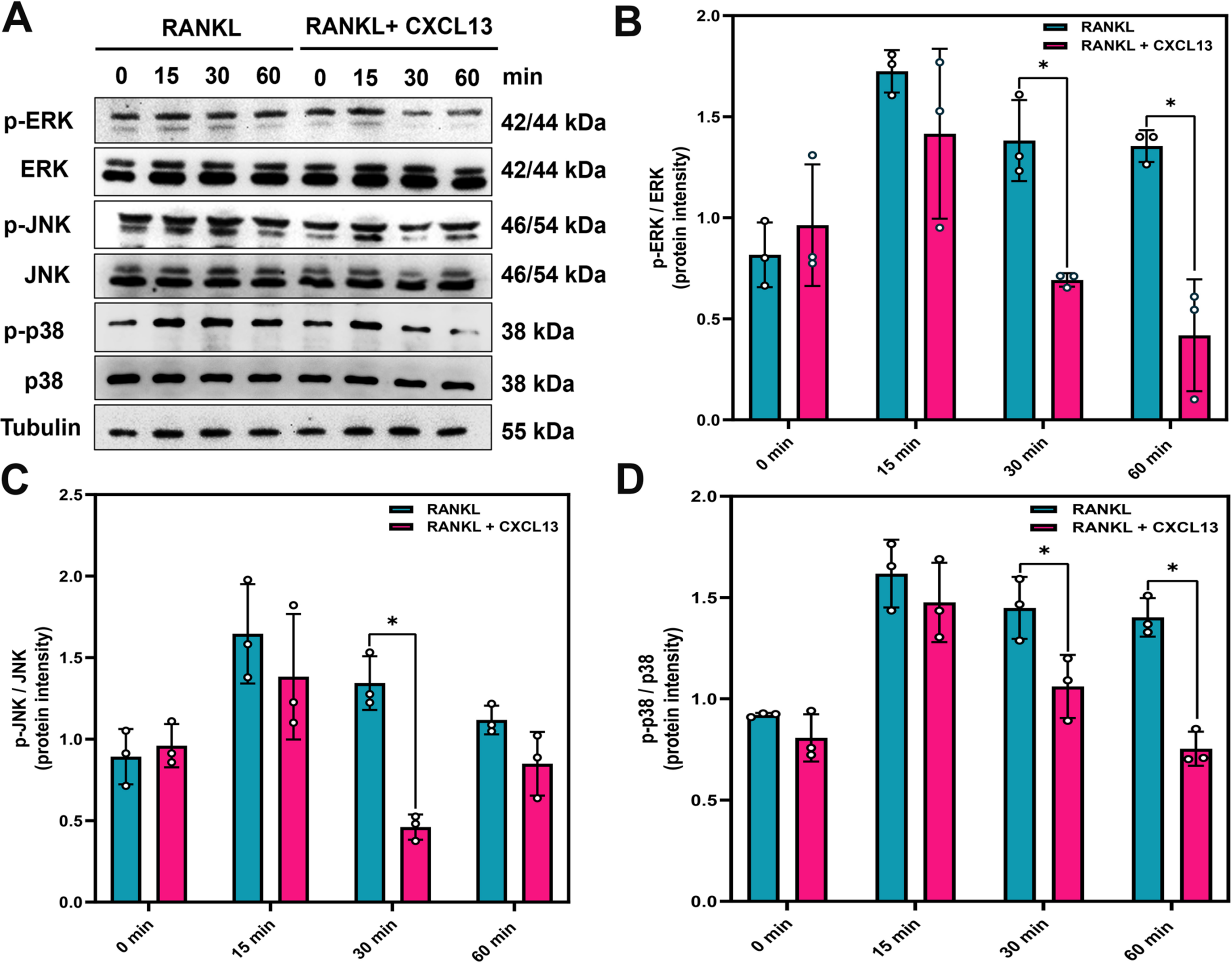
CXCL13 suppresses RANKL-induced activation of the MAPK signaling pathway. **(A)** RAW264.7 cells were treated with RANKL (50 ng/mL) alone or in combination with CXCL13 (0.5 ng/mL) for 0, 15, 30, or 60 minutes. MAPK pathway activation was evaluated via western blot, examining phosphorylated and total levels of ERK, JNK, and p38. Tubulin served as the internal loading control. **(B-D)** Band intensities were quantified using ImageJ. *p < 0.05 compared to the RANKL-treated group. Data represent biological replicates (n = 3). Statistical analysis was performed using one-way ANOVA followed by Tukey’s *post hoc* test.

**Figure 4:**
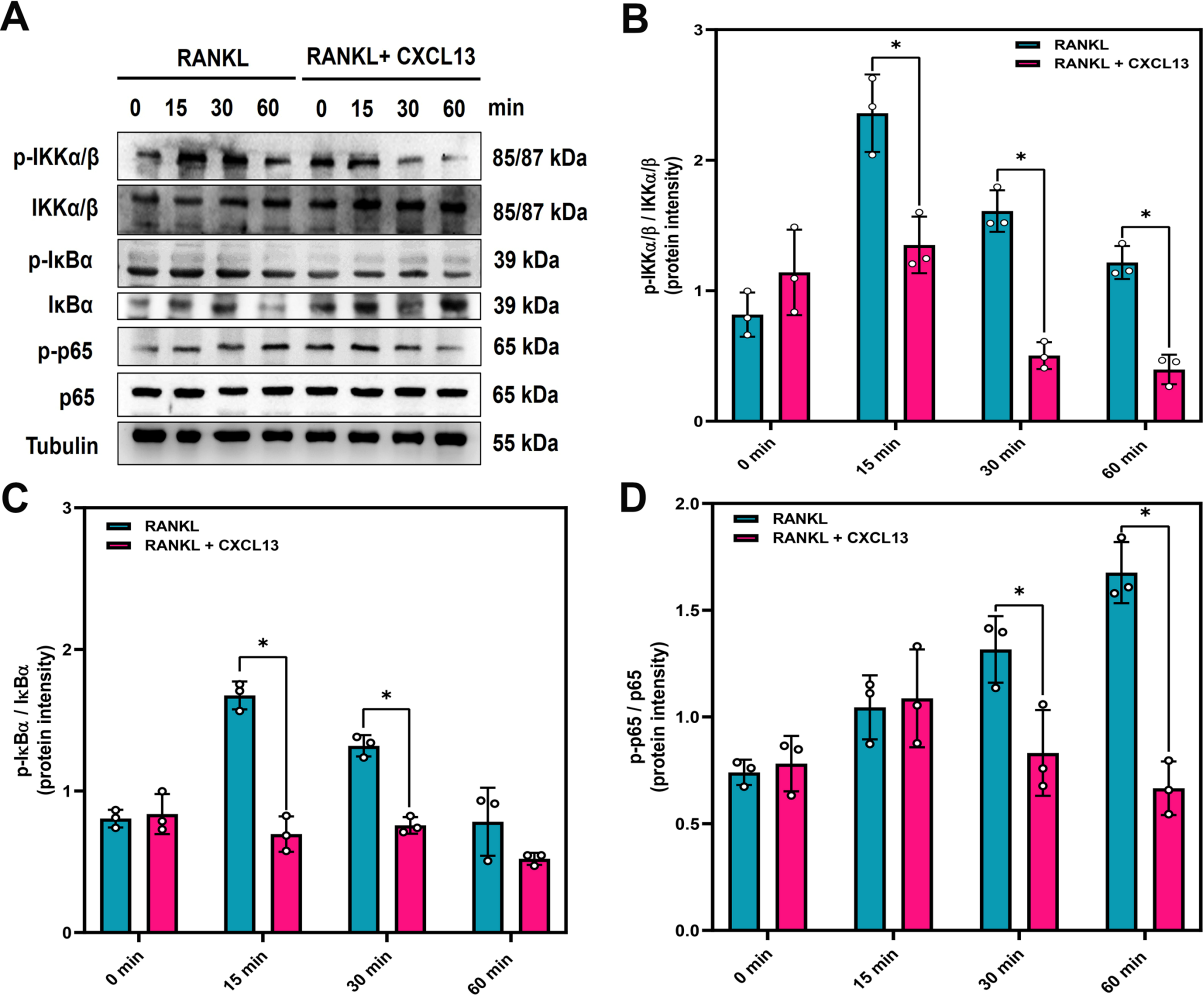
CXCL13 suppresses RANKL-induced activation of the NF-κB signaling pathway. **(A)** RAW264.7 cells were stimulated with RANKL (50 ng/mL) in the absence or presence of CXCL13 (0.5 ng/mL) for 0, 15, 30, or 60 minutes. NF-κB signaling components, including phosphorylated and total IKKα/β, IκBα, and p65, were analyzed by western blotting. Tubulin served as a control. **(B-D)** Quantitative analysis of protein levels was conducted using ImageJ software. *p < 0.05 compared to the RANKL-treated group. Data represent biological replicates (n = 3). Statistical analysis was performed using one-way ANOVA followed by Tukey’s *post hoc* test.

**Figure 5:**
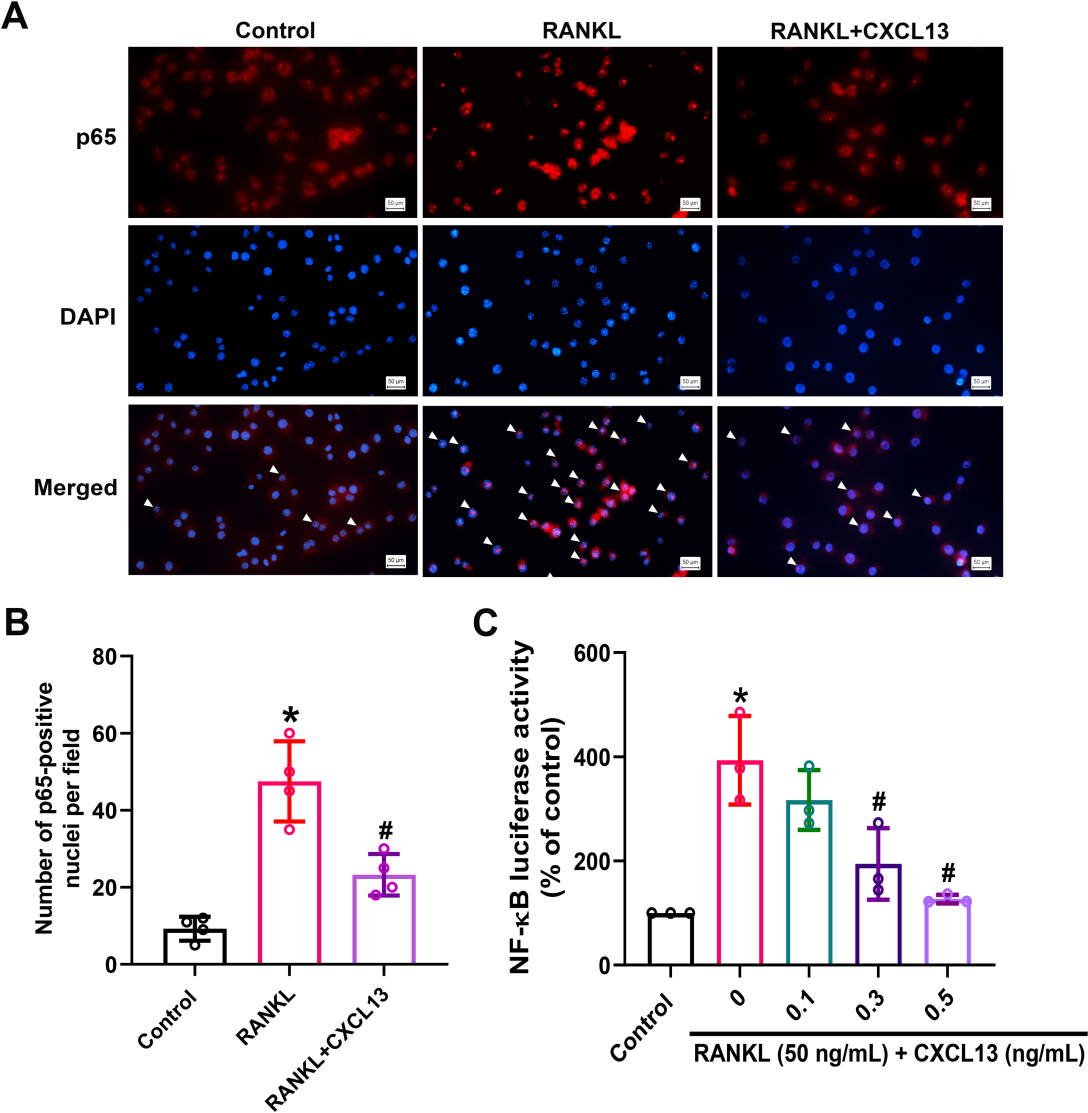
CXCL13 disrupts RANKL-induced p65 translocation to the nucleus. **(A, B)** Immunofluorescence staining revealed the distribution of the p65 subunit (red) and nuclei (DAPI, blue) after 4-hour RANKL treatment with or without CXCL13. Nuclear translocation was evaluated by measuring fluorescence overlap using ImageJ. **(C)** NF-κB luciferase reporter assay was conducted following transfection of RAW264.7 cells with the reporter plasmid and subsequent treatment with RANKL and CXCL13 at varying concentrations (0.1, 0.3, 0.5 ng/mL) for 24 hours. Transcriptional activity was quantified using a dual-luciferase assay. *p < 0.05 *vs*. control group; #p < 0.05 *vs*. RANKL-treated group. Data represent biological replicates (n ≥ 3). Statistical analysis was performed using one-way ANOVA followed by Tukey’s *post hoc* test.

RANKL stimulation triggers activation of the MAPK (ERK, JNK, and p38) and NF-κB signaling pathways, the latter involving IKKα/β phosphorylation, IκBα degradation, and p65 nuclear translocation, all of which are critical for osteoclast differentiation (36). To investigate whether CXCL13 modulates these pathways, we examined the phosphorylation status of MAPKs following RANKL stimulation. The results demonstrated that CXCL13 inhibited phosphorylation of ERK, JNK, and p38 (**Figure 3**). CXCL13 treatment also reduced phosphorylation of IKKα/β and effectively prevented IκBα degradation. Additionally, phosphorylation of the NF-κB p65 subunit was significantly reduced by CXCL13 treatment (**Figures 4A–D**). Immunofluorescence analysis demonstrated that RANKL stimulation markedly promoted the nuclear translocation of p65 in RAW264.7 cells. In contrast, the unstimulated group exhibited weaker nuclear p65 expression and more prominent cytoplasmic staining, consistent with its predominantly inactive state under basal conditions. Notably, CXCL13 treatment significantly attenuated RANKL-induced p65 nuclear localization (**Figures 5A, B**). Furthermore, RANKL stimulation markedly increased NF-κB luciferase activity compared to untreated controls (**Figure 5C**). Notably, CXCL13 treatment markedly reduced RANKL-induced NF-κB luciferase activity (**Figure 5C**). Together, these results demonstrate that CXCL13 effectively inhibits RANKL-induced activation of MAPK and NF-κB signaling pathways, leading to the suppression of osteoclast differentiation and function.

### CXCL13 competes with RANKL for binding to RANK, leading to the suppression of osteoclast differentiation

**Figure 6:**
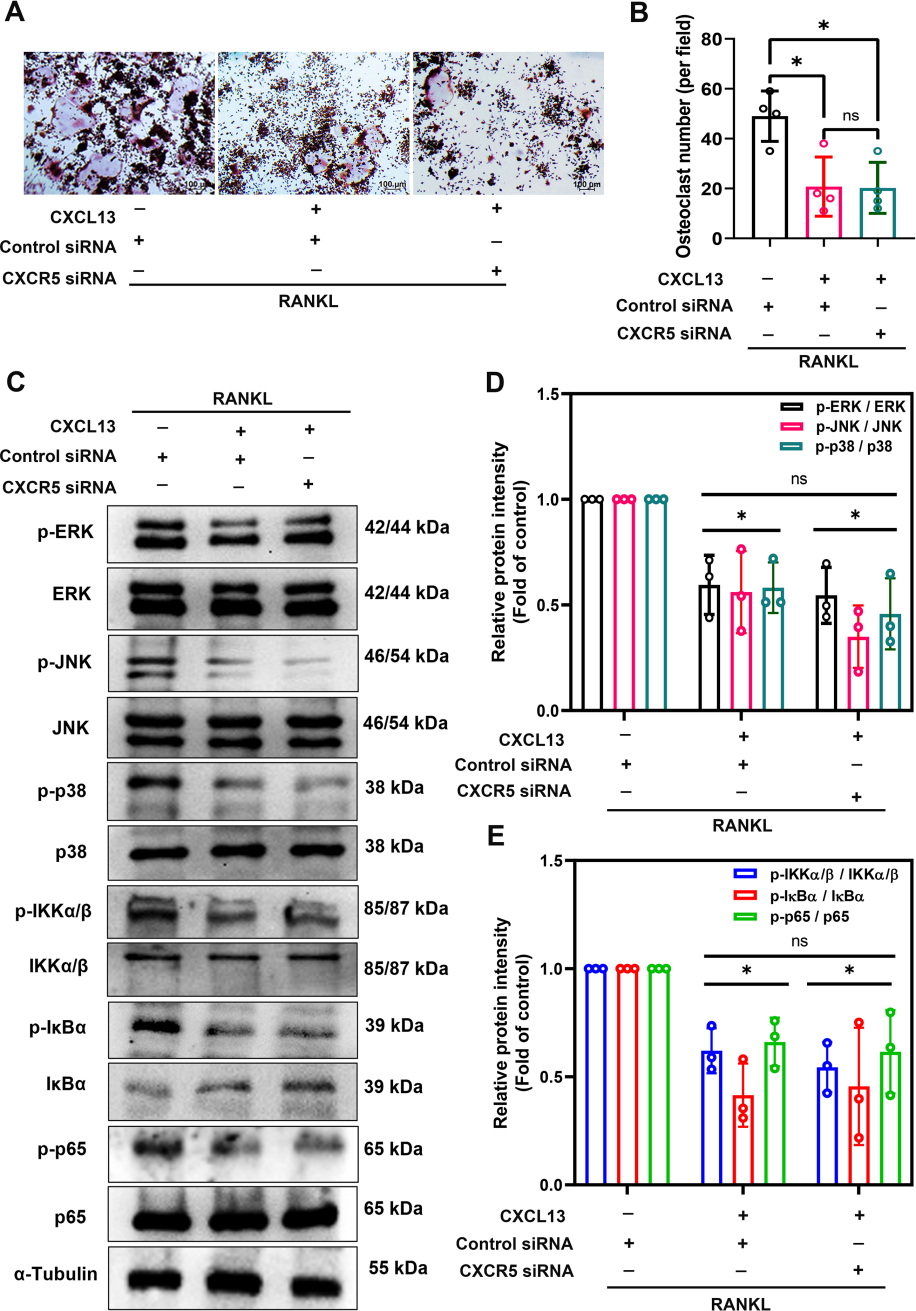
Inhibition of CXCR5 did not effectively reverse the inhibitory effect of CXCL13 on RANKL-induced osteoclastogenesis. RAW264.7 cells were transfected with CXCR5 siRNA before exposure to RANKL (50 ng/mL) for 5 days to induce osteoclast formation, followed by CXCL13 (0.5 ng/mL) treatment for 48 hours. **(A, B)** TRAP staining quantified osteoclast numbers and areas. **(C-E)** Western blot analysis measured the activation of MAPK and NF-κB signaling pathways, and band intensities were analyzed using ImageJ. *p < 0.05 compared to the RANKL-treated group. Data represent biological replicates (n ≥ 3). Statistical analysis was performed using one-way ANOVA followed by Tukey’s *post hoc* test.

CXCR5 is the canonical receptor for CXCL13 and is known to regulate diverse biological responses (22). To determine whether CXCL13’s effects on osteoclast differentiation are mediated through CXCR5, we silenced *Cxcr5* expression using siRNA during RANKL-induced osteoclastogenesis in the presence of CXCL13. TRAP staining revealed that CXCR5 knockdown failed to reverse the inhibitory effect of CXCL13 on osteoclast differentiation (**Figures 6A, B**). Western blot analysis demonstrated that CXCR5 silencing also did not affect CXCL13’s ability to suppress the phosphorylation of MAPKs and NF-κB signaling components (**Figures 6C-E**). These results suggest that the inhibitory effects of CXCL13 on osteoclastogenesis are independent of CXCR5.

**Figure 7:**
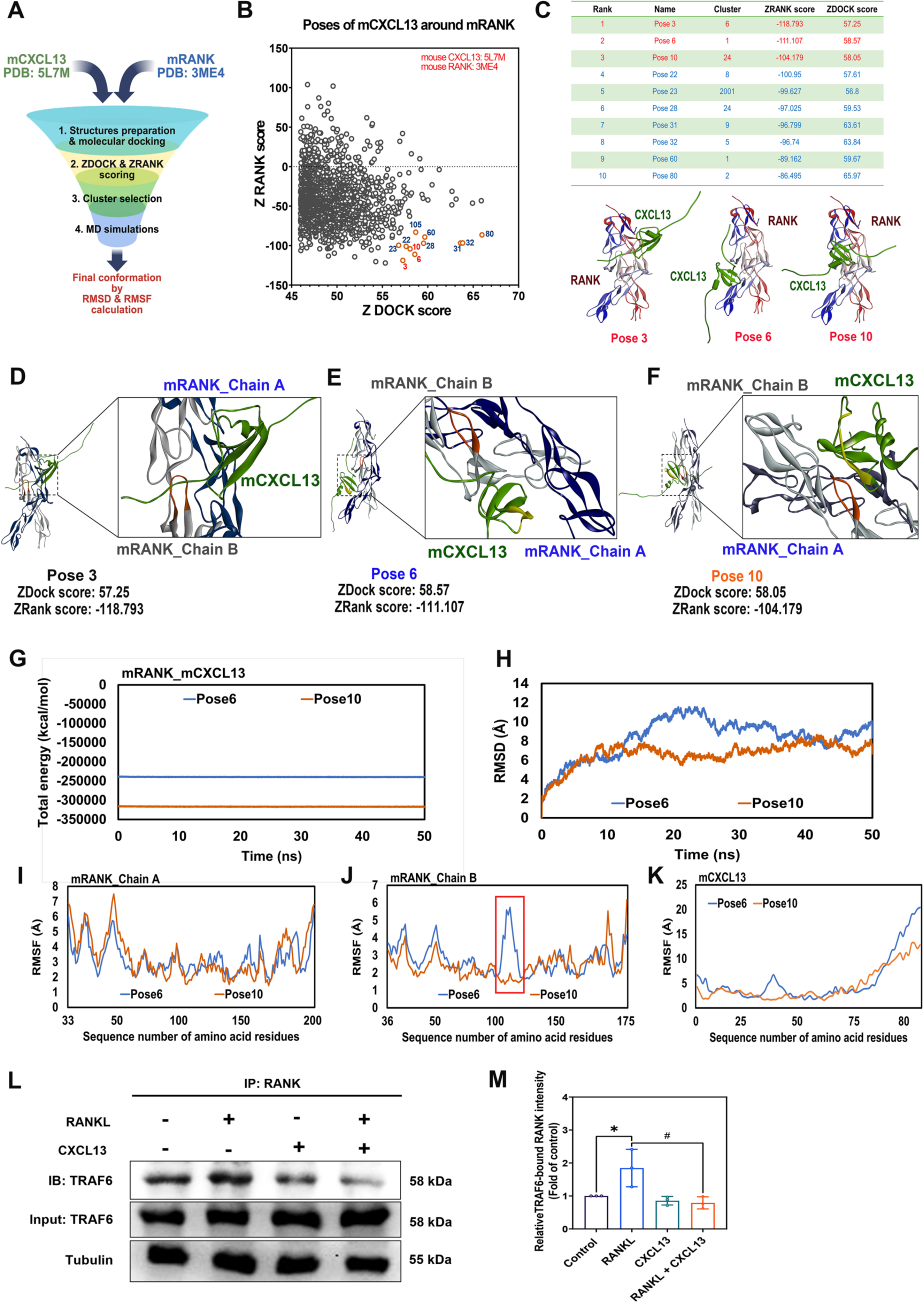
Predicted molecular interaction between CXCL13 and RANK. **(A)** The virtual screening process of macromolecular docking by Discovery Studio system. **(B, C)** Molecular docking identified multiple potential binding poses between CXCL13 and RANK. The top 10 poses were selected based on ZDOCK ranking scores, and representative docking conformations of poses 3, 6, and 10 are shown. **(D-F)** Detailed view of binding configurations for pose 3, pose 6 and pose 10. Orange indicates the amino-acid residues of the mRANK B-chain that form the stable interaction interface with mCXCL13. Blue represents the A-chain of mRANK, gray denotes the B-chain, and green highlights mCXCL13. **(G)** Calculated binding energies for selected poses. **(H-K)** Molecular dynamics simulations assessed the stability and flexibility of the CXCL13-RANK complexes, presenting RMSD and RMSF profiles. The red rectangle marks the critical interaction region in the RANK chain B that forms a stable interface with mCXCL13 in pose 10. **(L, M)** Visualization of dynamic interactions for both poses. **(K, L)** Co-immunoprecipitation and western blot were used to assess the interaction between RANK and TRAF6 under RANKL stimulation, with or without CXCL13 treatment. *p < 0.05 *vs*. control group; #p < 0.05 *vs*. RANKL-treated group. Data represent biological replicates (n ≥ 3). Statistical analysis was performed using one-way ANOVA followed by Tukey’s *post hoc* test.

Based on this, we hypothesized that CXCL13 may interact directly with the RANK receptor. To explore this possibility, molecular docking studies were conducted using Discovery Studio software. During the macromolecular docking analysis, virtual screening was performed using the structures of mRANK and mCXCL13 obtained from the Protein Data Bank (PDB). After structure preparation and docking with the ZDOCK platform, primary screening based on ZDOCK/ZRANK scores and pose clustering was conducted to narrow down the potential binding poses for subsequent molecular dynamics (MD) simulations and determination of the final conformation (**Figure 7A**). Among 2,000 predicted binding poses between CXCL13 and RANK (**Figure 7B**), poses 3, 6, and 10 showed the strongest binding affinities (**Figure 7C**). Although pose 3 exhibited a more favorable energy score compared with the other poses, we selected poses 6 and 10 for the MD simulation steps due to their clustering characteristics and overall reliability (**Figures 7D-F**). CXCL13 was predicted to bind to the extracellular domain of RANK with binding energies derived from pose 6 and pose 10 remaining stable throughout a 50-ns molecular dynamics simulation (**Figure 7G**). To evaluate the stability of these complexes, molecular dynamics simulations were conducted. Root mean square deviation (RMSD) analysis revealed that pose 10 maintained greater structural stability over time compared to pose 6 (**Figure 7H**). Root mean square fluctuation (RMSF) analysis further indicated reduced atomic mobility at the interface between chain B, rather than chain A, in the pose 10 complex, supporting its enhanced conformational stability (**Figures 7I-K**). Upon RANKL binding, TNF receptor-associated factor 6 (TRAF6) is recruited to the cytoplasmic domain of RANK, initiating activation of key pathways, such as MAPKs and NF-κB, which drive transcription of osteoclastogenic genes (37). A Co-IP assay demonstrated that RANKL markedly promoted the interaction between RANK and TRAF6, whereas co-treatment with CXCL13 substantially reduced TRAF6 recruitment to RANK (**Figures 7L, M**). These results indicate that CXCL13 inhibits RANKL-mediated osteoclastogenesis by blocking RANKL-RANK signaling, independent of CXCR5.

## Discussion

Chemokines are recognized as important regulators of osteoclast formation and activity. For example, CCL2 enhances osteoclast survival and bone-resorptive function (38), whereas CCL7 promotes osteoblast differentiation while reducing RANKL-induced osteoclast formation (39). Similarly, CXCL5 suppresses osteoclastogenesis in a dose-dependent manner via the p65 signaling pathway (40). However, the role of CXCL13 in osteoclast formation was previously unknown. Consistent with CCL7 and CXCL5, our study revealed that CXCL13 inhibits RANKL-induced osteoclast formation. CXCL13 also suppressed RANKL-induced MAPK and NF-κB signaling. Thus, chemokines act as both positive and negative regulators of osteoclastogenesis. Interestingly, we found that the inhibitory effect of CXCL13 is not mediated through its canonical receptor, CXCR5, but rather through direct interaction with RANK. Whether other chemokines share a similar mechanism requires further investigation.

Osteoclastogenesis and bone resorption are tightly controlled by the expression of osteoclast-specific genes. Among these, *Nfatc1* is a master mediator of osteoclast fusion and differentiation (41), while *Acp5, Ctsk*, and *Mmp9* are essential for the bone-resorptive functions of mature osteoclasts (42–45). Our data show that CXCL13 markedly downregulated the expression of these RANKL-induced genes in RAW264.7 cells, further supporting its inhibitory role in osteoclastogenesis. Mature osteoclasts were differentiated by treating RAW264.7 cells with RANKL for 5 days. CXCL13 reduced the viability of mature osteoclasts, as evidenced by a significant inhibit in the number and area of TRAP-positive multinucleated cells. CXCL13 treatment also disrupted F-actin ring formation, a marker of podosome belt integrity essential for bone matrix attachment and resorption in mature osteoclasts. Furthermore, CXCL13 stimulation increased cleaved caspase-3 expression in mature osteoclasts, indicating induction of apoptosis. Thus, CXCL13 not only inhibits osteoclastogenesis but also induces apoptosis in mature osteoclasts.

In our study, DMEM was used for routine maintenance of RAW264.7 cells because it supports optimal basal proliferation and viability during general culture (46). In contrast, α-MEM was specifically used during RANKL-induced osteoclast differentiation, as this medium contains higher levels of nutrients, including ascorbic acid and additional amino acids, which more effectively support osteoclast maturation and functional differentiation (47–49). Thus, the use of DMEM for RAW264.7 maintenance and α-MEM for osteoclastogenesis follows widely accepted protocols in the osteoclast biology field. At present, we are unable to perform additional experiments using human osteoclasts due to limitations in access to primary human samples and the technical constraints of our current model system. Nonetheless, we agree that validation in human osteoclasts would substantially strengthen the translational relevance of our findings. Although we did not perform bone resorption assays in this study, previous work from our group and others has shown a strong correlation between TRAP-positive multinucleated osteoclast formation and resorption pit activity in RAW264.7-derived osteoclasts (8, 50, 51). Consistent with this relationship, our TRAP staining results showed that RANKL robustly induced mature osteoclast formation, whereas CXCL13 treatment significantly reduced this maturation.

In this study, we identified a novel role of CXCL13 as a negative regulator of RANKL-induced osteoclast formation. Similarly, Osteoprotegerin (OPG)-a well-established inhibitor of osteoclastogenesis-functions as a soluble decoy receptor for RANKL and is primarily produced by osteoblasts and stromal cells (52). In contrast, previous studies have detected OPG expression in osteoclasts differentiated from mouse bone-marrow-derived cells (BMDCs) and suggesting a potential autocrine regulatory role during late osteoclast differentiation (53). Because our osteoclast differentiation model does not include osteoblasts or stromal cells, cytokine-mediated OPG induction (e.g., via IL-4 or IL-13) (54) cannot occur in this system, further supporting that the inhibitory effect of CXCL13 is independent of osteoblast-derived OPG. Whether CXCL13 suppresses osteoclastogenesis indirectly through OPG induction is an important consideration. Although this possibility cannot be excluded, it remains to be fully elucidated, and future studies will be directed toward investigating this potential mechanism.

The MAPK and NF-κB signaling pathways are well-established cascades activated by RANKL-RANK interaction, crucial for osteoclast differentiation, activation, and survival (55–57). The MAPK family, comprising ERK, JNK, and p38, regulates transcriptional and post-transcriptional events critical for osteoclast function. Here, we demonstrated that CXCL13 markedly suppressed RANKL-induced phosphorylation of ERK, JNK, and p38 in RAW264.7 cells. NF-κB pathways are well-characterized downstream signaling cascades activated upon RANKL–RANK interaction and are essential for osteoclast differentiation, activation, and survival (44–46). Activation of the NF-κB pathway, particularly through phosphorylation and nuclear translocation of the p65 subunit, is crucial for initiating osteoclastogenic gene expression. We found that CXCL13 inhibited RANKL-induced phosphorylation of IKKα/β, IκBα, and p65, as well as p65 nuclear translocation and NF-κB luciferase activity. These results suggest that the inhibition of the NF-κB pathway is critical for CXCL13’s suppression of osteoclastogenesis. Similar findings have been reported for other compounds: Ugonin L suppresses osteoclastogenesis by reducing MAPK and NF-κB signaling (8), a caffeic acid derivative (MPMCA) inhibits these pathways to reduce osteoclast differentiation (58), and melatonin diminishes osteoclast function and osteolytic bone metastasis via MAPK and NF-κB inhibition (59).

Molecular docking analysis, combined with Co-IP assays, suggests that CXCL13 interferes with the interaction between RANKL and RANK, thereby attenuating downstream signaling pathways involved in osteoclastogenesis. A total of 2,000 binding poses between CXCL13 and RANK were generated during docking analysis, and the top 10 poses were further evaluated based on binding affinity and interaction stability. Among these, poses 6 and 10 emerged as the most promising candidates, exhibiting high binding energies and favorable spatial orientations within the ligand-binding domain of RANK. To provide a more comprehensive overview of the ZDOCK results, we include a table summarizing the ZDOCK/ZRANK scores together with the number of poses within each cluster, which also serves as a criterion for evaluating pose accuracy. Notably, the conformation of RANK-pose 3 complex differed from that of poses 6 and 10. In pose 3, CXCL13 primarily binds to chain A of RANK, resulting in its cluster containing only a single pose within the top ten rankings. In contrast, poses 6 and 10 were selected for MD simulations due to their clustering characteristics and overall reliability (**Figures 7C-F**).

Co-IP assays confirmed that CXCL13 substantially reduced TRAF6 recruitment to RANK. These findings support the hypothesis that CXCL13 may directly interact with RANK, potentially competing with RANKL for receptor binding. Previous molecular docking studies have explored similar mechanisms. For example, a selective RANKL-binding compound, S3-15, was found to target soluble RANKL and effectively block its interaction with RANK, demonstrating anti-osteoporotic efficacy *in vivo* without immunosuppression (60). Similarly, molecular docking and dynamics simulations have revealed that the bisphosphonate zoledronic acid interacts with RANKL, providing mechanistic insights into its anti-resorptive activity (61). Although competitive binding assays were not performed in the present study, future investigation directly comparing the binding affinities of CXCL13 and RANKL for RANK will be important for elucidating the precise mechanism underlying their interaction.

**Figure 8:**
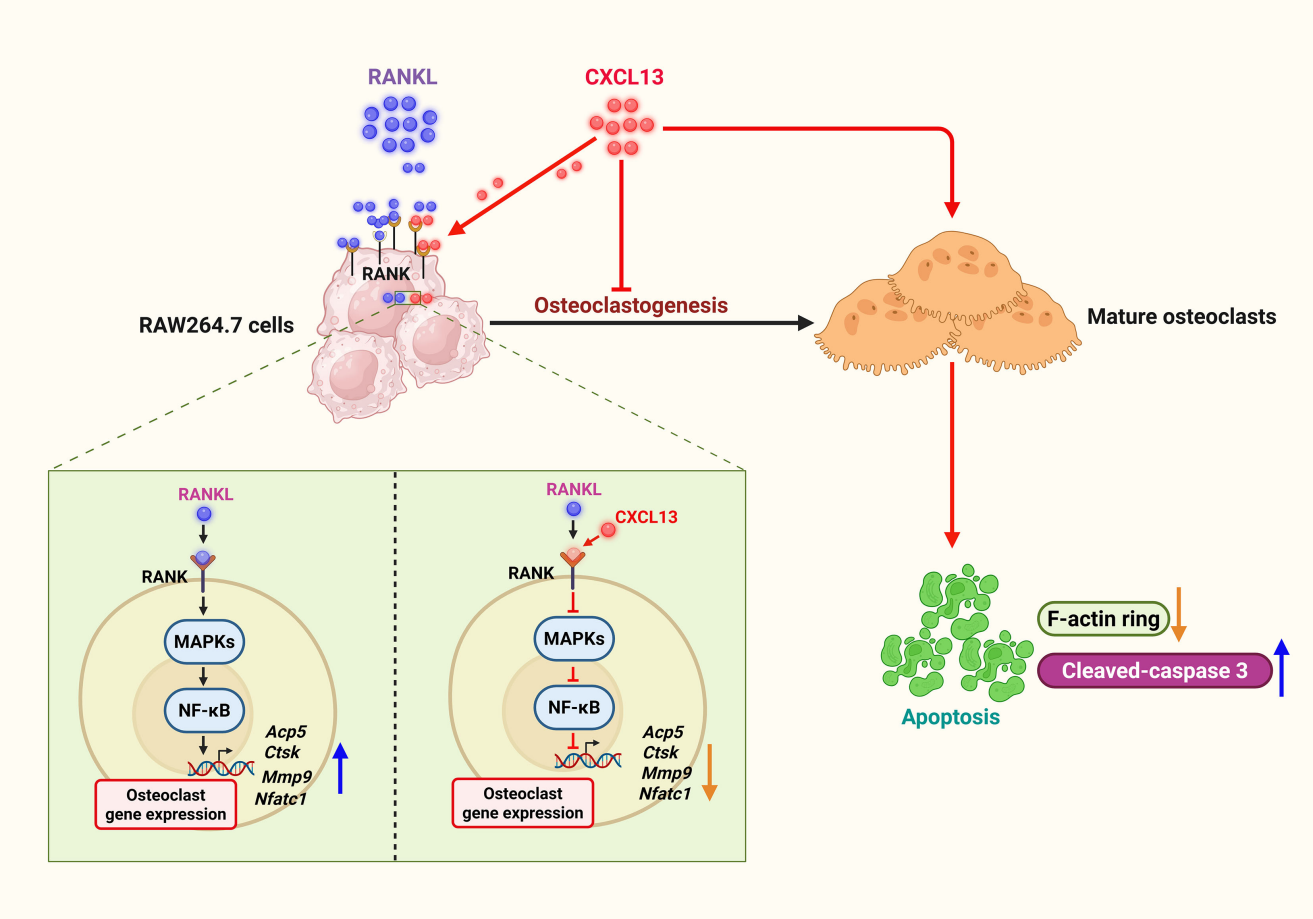
Schematic illustration of signaling pathways involved in CXCL13-inhibited osteoclast formation. CXCL13 impedes RANKL-induced osteoclastogenesis by downregulating the activation of MAPK and NF-κB signaling pathways. CXCL13 also directly induces apoptosis in mature osteoclasts. Notably, CXCL13 restricts RANKL-RANK signaling, but its inhibitory effects are mediated independently of its canonical receptor, CXCR5.

Our study shows that CXCL13 suppresses RANKL-induced osteoclastogenesis *in vitro* by inhibiting MAPK and NF-κB signaling pathways and promoting apoptosis in mature osteoclasts. Importantly, CXCL13 limits RANKL–RANK signaling independently of its canonical receptor, CXCR5, highlighting its distinct mechanism of action in regulating osteoclast differentiation (**Figure 8**).

## Data Availability

The original contributions presented in the study are included in the article/supplementary material. Further inquiries can be directed to the corresponding author.
